# Histone variants: key regulators of inflammation in cell dedifferentiation and transdifferentiation

**DOI:** 10.3389/fimmu.2025.1619100

**Published:** 2025-06-27

**Authors:** Manlio Vinciguerra, Desislava K. Tsoneva

**Affiliations:** Department of Translational Stem Cell Biology, Research Institute, Medical University Varna, Varna, Bulgaria

**Keywords:** histone variants, inflammation, differentiation, stem cells, epigenetics

## Abstract

Histone variants are specialized isoforms of histone proteins that play crucial roles in regulating chromatin structure and function, influencing transcription, DNA repair, and cell cycle progression. Their dynamic incorporation into nucleosomes impacts gene expression and cellular identity, particularly in the context of inflammation during cell dedifferentiation and transdifferentiation. This mini-review provides a comprehensive overview of the role of histone variants in these processes, highlighting their significance in modulating inflammatory responses and cellular plasticity. We discuss mechanisms by which histone variants influence chromatin architecture and gene regulation, the interplay between histone variants and inflammatory pathways, and the specific roles of key histone variants such as H3.3, H2A.Z, and MacroH2A in dedifferentiation and transdifferentiation. Additionally, we explore the potential therapeutic implications of targeting histone variants to modulate inflammation and cellular plasticity in diseases like cancer and chronic inflammatory conditions. By summarizing existing knowledge and identifying gaps in understanding, this review underscores the importance of histone variants in inflammation-related cell plasticity and suggests future research directions further elucidating their roles and therapeutic potential.

## Introduction

Histone variants are isoforms of the linker and core histone proteins that differ from canonical histones in their amino acid sequences and post-translational modifications (PTMs), leading to unique genomic locations and functions (1, 2). The dynamic incorporation of histone variants into nucleosomes significantly impacts nucleosome stability and creates functionally distinct chromatin domains essential for various cellular processes (2–4). In inflammation, histone variants modulate the inflammatory response by altering chromatin structure and gene expression. For example, accumulation of the histone variant H3.3 at specific loci is associated with increased transcriptional activity, contributing to inflammatory processes (5). In senescent cells, the loss of canonical histones H3 and H4 and the accumulation of selected histone variants lead to the secretion of pro-inflammatory factors, establishing a pro-inflammatory environment that drives chronic inflammation and tissue dysfunction (5). Cell dedifferentiation, the process by which specialized cells revert to a more primitive state, is closely linked to chromatin dynamics and histone variant incorporation. This involves the downregulation of key genes and the upregulation of genes typically suppressed in differentiated cells (6). Histone variants such as H3.3 and H2A.Z are associated with transcriptional regulation at active genes and can reset epigenetic states critical for dedifferentiation (7). Transdifferentiation, where one differentiated cell type converts into another, also involves histone variants. For instance, manipulating α cell-specific transcription factor Arx or ablation of Pdx1 in β cells can induce transdifferentiation of β cells into α cells and vice versa (8–10). The dynamic behavior of linker histones, such as H1 variants, influences chromatin compaction and accessibility, impacting both dedifferentiation and transdifferentiation (7). Inflammation-induced changes in histone variants and their PTMs are crucial in regulating gene expression during inflammation and cell dedifferentiation. These modifications can activate or repress gene expression, modulating inflammatory responses and contributing to the pathogenesis of inflammatory diseases (5, 11).

**Figure 1:**
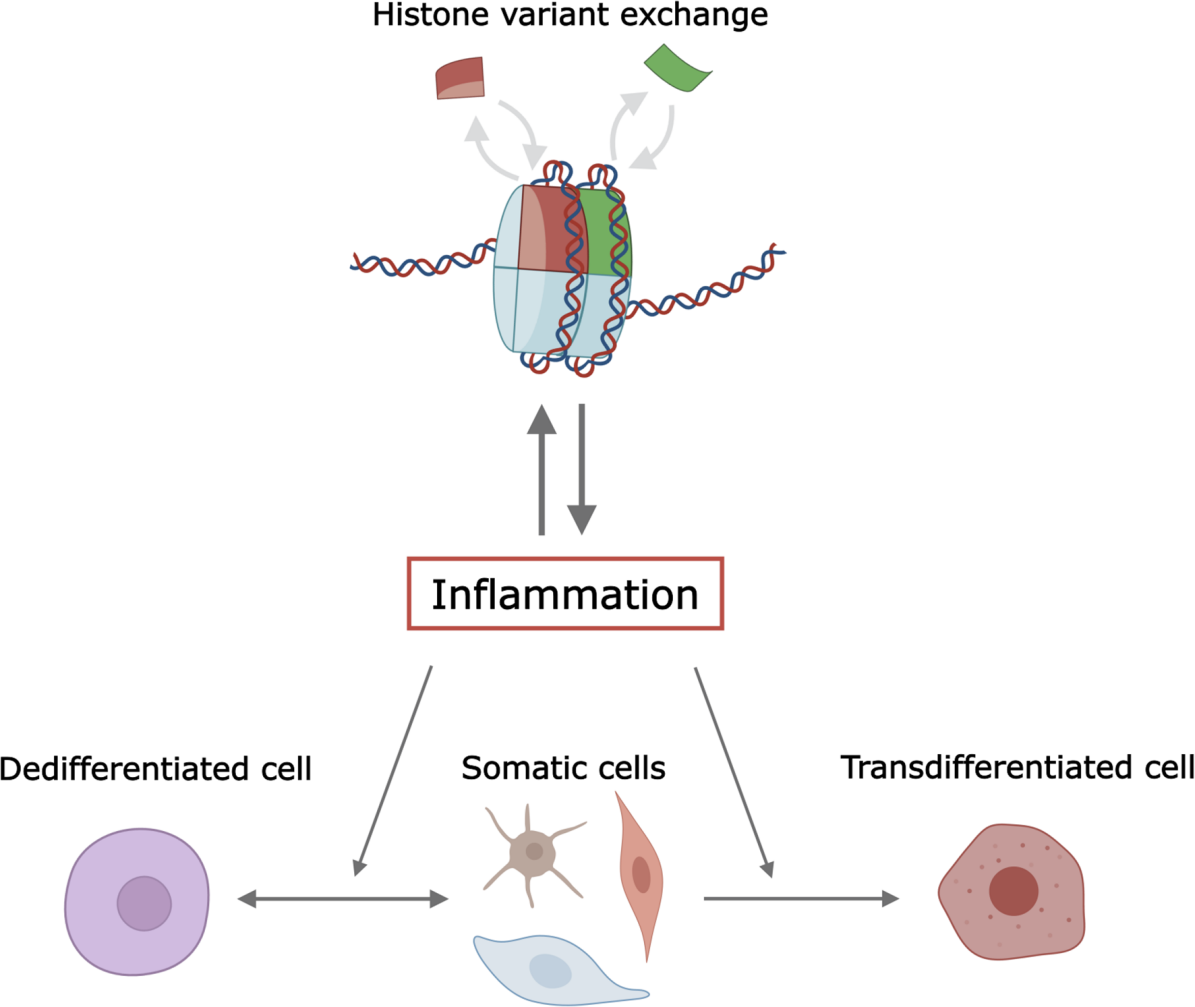
Illustration of the interplay between histone variants, inflammation, and cellular plasticity. The figure is created in Inkscape and partially with Biorender. 219.

This mini-review explores the role of histone variants in inflammation during cell dedifferentiation and transdifferentiation. We discuss mechanisms by which histone variants influence these processes, the interplay between histone variants and inflammatory pathways, and the potential therapeutic implications of targeting histone variants in inflammation-related cellular plasticity (**Figure 1**). Understanding these mechanisms is vital for developing targeted therapies for diseases characterized by aberrant inflammation and cellular plasticity.

## Histone variants and inflammation in epigenetic remodeling

Histone variants, first described in 1977 (12), are critical chromatin components, differing from canonical histones (H1, H2A, H2B, H3, H4) in their amino acid sequences and PTMs. All canonical histones have variants, which contribute to structural and physical diversities to the nucleosome core particle (13). Since 2017, histone variants have followed a unified phylogeny-based nomenclature (14). In contrast to canonical histones being encoded by multiple genes, histone variants are usually encoded by one or a few genes, and some are produced in specific tissues. The differences from canonical histones result in unique histone variant genomic localization and functions, enhancing the complexity of chromatin architecture and enabling specialized roles in gene regulation, DNA repair, and cell cycle progression (3, 4, 15). Unlike the canonical histone mRNAs, the histone variant mRNAs are poly-adenylated, and the proteins are produced in cell cycle- and replication-independent manners. Therefore, while canonical histones are synthesized primarily during DNA replication, histone variants are expressed throughout the cell cycle and can replace canonical histones during various biological processes (16). Among the core histones, the H2A family has the greatest number of known variants, exhibiting the highest sequence divergence (17), with the “short H2A variants”, which lack a C-terminal tail, being the most divergent (5). The variants of H2A, primarily H2A.X, H2A.Z, and macroH2A, are well-established participants in the genome integrity protection (18). H3 variants, including H3.3, centromeric H3 variant (cenH3, also called CENPA in humans) play significant roles in active transcription and regulatory regions (19). Histone H2B and H4 are among the slowest evolving proteins with functional variants often tissue-restricted, such as the testis, or species-restricted, such as Trypanosoma for H2B and H4 variants, respectively (20, 21). Although less studied, histone H1 variants also contribute to chromatin structure and gene regulation (22).

Inflammation is a first responder against injury and infection and is also critical for the regeneration and repair of tissue after injury. Immune and non-immune cells directly respond to the local inflammatory cues and can undergo phenotypic and differentiation potential modifications (i.e. cell differentiation) or even cell lineage transitions (i.e. transdifferentiation) in physiological and pathological settings (see next section). In this respect, histone variants create unique chromatin states that influence transcriptional regulation (23) and therefore, cellular processes involving gene expression switch such as embryonic development, stem cell lineage commitment, somatic cell reprogramming, inflammatory signaling, and aging (24–26).

Specific examples include the histone variants H2A.J, H2AX, and macroH2A. H2A.J was shown to accumulate in the chromatin of senescent cells with persistent DNA damage caused by replication stress, phosphorylated H2AX (γ-H2AX)-marked double-strand DNA breaks, or RAS oncogene (27). Downregulation of H2A.J impedes inflammatory gene expression, including the expression of senescent-associated secretory proteins (SASP), while overexpression induces the expression of those genes (27). γ-H2AX is a well-established marker of double-strand DNA breaks, connecting DNA damage and chronic inflammation. Region-unspecific H2AX phosphorylation was observed following viral DNA replication onset, but not following nonreplicating virus infection (28). MacroH2A is a unique histone variant of H2A with its nonhistone region (NHR) contributing to promoter-specific repression of gene expression due to its large size (29). Such macroH2A-mediated gene expression inhibition is reflected in inflammatory signaling modulation. MacroH2A histones hinder the chromatin remodeling capacity of the SWI/SNF complex on macroH2A-containing nucleosomes and block specific NF-κB binding to nucleosomes (29), causing deregulated inflammatory responses by repressing expression of several pro-inflammatory genes and activation of inflammatory T cells (30). Conversely, NAP1 chaperone-mediated incorporation of H2A.Z in the nucleosome elicits a proinflammatory response by nucleosome remodeling at the TNFα promoter (31).

## Cell differentiation and transdifferentiation: role for histone variants

Cell dedifferentiation involves the loss of specialized cellular identity and regression to a less differentiated state. It is a transient process by which cells become less specialized and return to an earlier cell state within the same lineage. Dedifferentiation implies an increase in cell potency, meaning that, following dedifferentiation, a cell may possess the ability to re-differentiate into more cell types than it did before dedifferentiation. On the other hand, transdifferentiation, also known as lineage reprogramming, is an uncommon process in which one mature somatic cell is transformed into another mature somatic cell without undergoing an intermediate pluripotent state or progenitor cell type (32, 33). Transdifferentiation is a type of metaplasia, which includes all cell fate switches, including the interconversion of stem cells. While several examples of transdifferentiation in invertebrates and in amphibians have been reported (34), the best example in humans and mice is the spontaneous fate switch of pancreatic α-cells into β-cells, which has been demonstrated for both healthy and diabetic pancreatic islets (35).

The dedifferentiation process is characterized by several mechanisms: 1. reduction in expression of lineage-specific genes, including essential transcription factors (TFs) and metabolic genes. In this respect, Hikichi et al. have shown that, of the large number of TFs expressed in a neural-lineage cell line, only a subset of TFs, when overexpressed, strongly interfered with the dedifferentiation triggered by the procedure to generate human induced pluripotent stem cells (iPSCs) (36). Among these TFs, ZBTB12 is a prominent molecular barrier to dedifferentiation in human iPSCs (37). Another example is shown by the dedifferentiation induction of β cells dedifferentiation following the deletion of the TF IRE1α, which results in the prevention of type 1 diabetes (38); 2. Activation of genes suppressed in normal differentiated cells and progenitor cell genes: almost any differentiated cell, in health and disease, can be returned to its pluripotent state by activating specific signaling pathways or by expressing the appropriate transcription factors (39). This can occur, for instance, through somatic reprogramming using Yamanaka factors (40), or by activation of the Wnt/β-catenin signaling pathway in epidermal cells (41) and endothelial cells (42) for regeneration; 3. Inflammation, oxidative stress, ER stress, and hypoxia contribute to dedifferentiation. IL-1β, IL-6, and TNFα have been shown to promote β-cell dedifferentiation in cultured human and mouse islets, with IL-1β being the most potent one of them (43, 44). Inflammation can also induce cancer dedifferentiation, as in metastatic melanoma (45). Oxidative and ER stresses reduce the functionality of endocrine cells by stimulating their de-/trans-differentiation through the loss of transcription factors critical for cell development, maturity, and regeneration, as observed in β-cells and thyrocytes (46, 47). As for hypoxia, a long-term hypoxic state maintains dedifferentiation in fetal cardiovascular progenitor cells through the Wnt/β-catenin signaling pathway (48), and favors transdifferentiation in a variety of cell types, including epithelial-to-mesenchymal (49), renal tubular cells into myofibroblasts (50), and pulmonary arterial endothelial cells into smooth muscle cells (51).

Histone variants significantly influence dedifferentiation and transdifferentiation by altering chromatin dynamics and gene expression. Incorporation of variants like H3.3 and H2A.Z at active genes is associated with transcriptional activation and resetting of epigenetic states vital for dedifferentiation and transdifferentiation (52–54). The histone variant H2A.Z is noteworthy for marking the 5’ ends of both active and inactive genes in euchromatin, influencing gene expression patterns during inflammation and cellular reprogramming (55) by enhancing the access of transcription factors to genes important for pluripotency (56). In response to vascular injury such as a myocardial infarction, vascular smooth muscle cells (VSMC) in the neointima undergo phenotypic switching from a differentiated to a dedifferentiated state, characterized by a significant reduction in contractile gene expression (57). H2A.Z occupies genomic regions near VSMC marker genes, and its occupancy is decreased in VSMCs undergoing dedifferentiation (58); its *in vivo* overexpression rescues injury-induced loss of VSMC and phenotypic switching from a dedifferentiated to a differentiated state, characterized by a significant reduction in contractile gene expression (57). H2A.Z occupies genomic regions near VSMC marker genes, and its occupancy is decreased in VSMCs undergoing dedifferentiation (58); its *in vivo* overexpression rescues injury-induced loss of VSMC and neointima formation (58). Histone variant H3.3B has also been implicated in the phenotypic transition of VSMCs, as well as in vascular inflammation in aortic dissection (59). Knock-down of H3.3 at the early stage of transdifferentiation - induced by forced expression of Scl, Lmo2, Runx1, and Bmi1 - gave rise to greater induced hematopoietic progenitor cells derived from mouse embryonic fibroblasts (54). The levels of histone variant H1.0 correlate with tumor cell differentiation and patient survival. Silencing H1.0 favors self-renewal, indicating that H1.0 helps maintain the differentiated state and may contribute to dedifferentiation (60). Linker histone B4, a variant of H1, is enriched at promoters of reactivated pluripotency genes and is required for pluripotency gene reactivation in oocytes (61), and transdifferentiation of pigmented epithelial cells *in vivo* (62).

## MacroH2A histone variants, dedifferentiation and inflammation

MacroH2A histone variants represent a useful proof-of-concept to illustrate the role of histone variants at the crossroad between dedifferentiation and inflammation. Its above-mentioned NHR domain protrudes from the compact structure of the nucleosome, likely affecting the function and organization of the surrounding chromatin (63). Variants of the macroH2A family, like macroH2A1 (coded by *H2AFY* gene) and macroH2A2 (coded by *H2AFY2* gene) regulate gene expression important for differentiation, stem cell reprogramming and tumor suppression. They can inhibit reprogramming by maintaining repressive chromatin states (60, 64–66). In fact, macroH2A’s role as a barrier to cellular plasticity might predict a possible role in preventing cancer, which can be considered a state of dedifferentiation (67–70). The levels of both macroH2A1 (and its exon splicing variants macroH2A1.1 and macroH2A1.2) and macroH2A.2 are strongly predictive of survival from numerous cancer types (71–81). MacroH2A-depleted low-malignancy melanoma exhibited enhanced tumor growth, cell motility, and metastasis, whereas its overexpression in highly malignant cells had the opposite effects (72). Moreover, in macroH2A-depleted melanoma, single-cell and spatial transcriptomics identified increased dedifferentiation in the tumor compartment, accumulation of cancer-associated fibroblasts and immunosuppressive monocytes, and depletion of functional cytotoxic T cells (82), resulting in tumor-infiltrating immune cell dysfunction and accelerated tumor growth and invasiveness.

## Histone variants, inflammation, and cell plasticity

It has been established that inflammation, an integral part of the regenerative response that entails cytokine production by innate and adaptive immune cells, can trigger cell dedifferentiation or transdifferentiation (83–86). In turn, inflammatory cytokines such as TNF-α and IL-6 can modulate the expression and incorporation of histone variants, altering chromatin accessibility and transcriptional activity (60). MacroH2A histones mediate gene expression in response to pro-inflammatory signals (87–90). In the macroH2A-depleted melanoma model, macroH2A-deficient cancer-associated fibroblasts display increased myeloid chemoattractant activity as a consequence of hyperinducible expression of inflammatory genes, which was enforced by increased chromatin looping of their promoters to enhancers that gain H3K27ac (82). In high-fat diet-induced obesity models, macroH2A1 isoforms regulate metabolic health and tissue inflammation (91, 92). At the genomic level, macroH2A1.2 and macroH2A2 regulate the inflammatory-induced transcriptional response of cancer cells by affecting enhancer-promoter contacts, therefore, modulating enhancer activity and sensitivity to inflammatory cytokines (93). Thanos and coworkers’ earlier work has shown that silencing macroH2A regulates inflammatory cytokine IL-8 transcription (87). Other histone variants-related mechanisms interact too with the inflammatory process: for instance, H2A.Z nucleosomes at type I interferon (IFN)-stimulated gene (ISG) promoters modulate the biological response to IFN (94); H3.3 phosphorylation amplifies inflammatory stimulation-induced transcription (95) and induces proatherogenic gene expression (96); loss of the H3.3 chaperone DAXX in hematopoietic precursors leads to inflammation (97). The mRNA expression levels of both H2A.Z and H3.3 were found elevated in peripheral blood mononuclear (PBMC) cells of patients with rheumatoid arthritis (98). H3.3 has been identified as a crucial epigenetic regulator of the innate immune system. Following inflammatory stimuli, H3.3 is phosphorylated at its S31 site, promoting gene transcription at activated genes (95, 99). H3.3 has also been shown to exhibit a silencing function on viral genomes through H3.3-dependent chromatinization (100–102), therefore, modulating anti-viral immunity. Deletion of H3.3 in hematopoietic stem progenitor cells (HSPCs) results in a loss of adult homeostatic hematopoiesis, myeloid lineage bias, and premature HSC exhaustion (103). The complex interplay between histone variants and inflammation affects cellular plasticity, contributing to disease progression. Moreover, the relationship with inflammation appears to be bidirectional. It is well established that very high circulating levels of histones, including histone variants, induce systemic inflammation, sepsis-like symptoms, and multi-organ injury in animal models and in patients (104–107). Intracellularly, histone variants regulate inflammatory responses by influencing pro-inflammatory gene expression, while inflammation alters histone variant expression and incorporation, impacting chromatin dynamics. Understanding this interplay is crucial for elucidating the mechanisms underlying dedifferentiation and transdifferentiation in inflammatory contexts.

## Discussion

Histone variants play a pivotal role in modulating inflammation during cell dedifferentiation and transdifferentiation by influencing chromatin structure and gene expression (**Figure 1**). Key variants such as H3.3, H2A.Z, and macroH2A are crucial in altering chromatin accessibility and transcriptional regulation, thereby modulating cellular plasticity. These insights present potential therapeutic avenues, including targeting specific histone variants to control inflammation and cellular reprogramming in diseases like cancer and diabetes. Despite these advancements, challenges persist in understanding the complex interplay between histone variants and other chromatin modifiers, as well as their context-dependent effects. Future research should aim to elucidate the precise mechanisms by which histone variants influence inflammation and cellular plasticity, utilizing advanced techniques like single-cell sequencing and CRISPR-based epigenome editing. Non-coding RNAs, such as lncRNAs and miRNA, are key players in the inflammatory response (108) and in cell reprogramming (109), respectively. The functional interactions between histone variants and non-coding RNAs remain mostly unexplored. Examples of functional interactions between non-coding RNA and histone variants include the conditional deletion of lncRNA Xist that disrupts histone macroH2A localization during X chromosome inactivation (110), and the lncRNA LHX1-DT-dependent regulation of cardiomyocyte differentiation through H2A.Z (111). Addressing systematically these epigenetic cross-talks and regulatory mechanisms will enhance our ability to develop targeted therapies that leverage the regulatory potential of histone variants in inflammation-related cellular plasticity processes.
